# Quick Three-Dimensional Transesophageal Echocardiography of Left Atrial Appendage (LAA) Anatomy Using the LAA Multiview Technique

**DOI:** 10.1016/j.case.2023.08.007

**Published:** 2023-10-11

**Authors:** Alan Vainrib, Muhamed Saric

**Affiliations:** Department of Cardiology, NYU Langone Health, New York, New York

**Figure:**
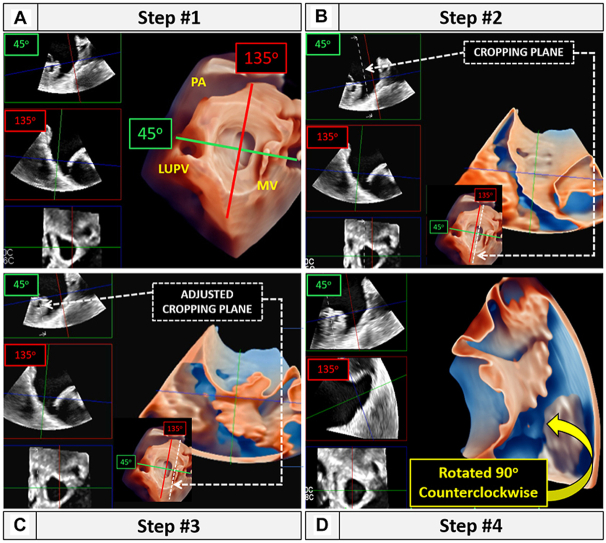

We are writing to provide an essential update to a recently published article in *CASE* titled “Left Atrial Appendage Tilt-Up-and-Turn-Left Maneuver: A Novel Three-Dimensional Transesophageal Echocardiography Imaging Maneuver to Characterize the Left Atrial Appendage and to Improve Transcatheter Closure Guidance.”^1^

We have now developed a simplified version of the above three-dimensional (3D) transesophageal echocardiography (TEE) maneuver, which minimizes the number of image rotation and cropping steps. We have termed this simplified version the 3D left atrial appendage (LAA) multiview (LAAM) technique. This approach will work with the current latest generation of Philips Ultrasound Systems and would need to be modified if used by other platforms.

The key feature of the LAAM technique is its ability to visualize the LAA anatomy by 3D TEE in only 4 steps (Figure 1, Video 1). It is based on the notion that the long axis of the body of LAA is typically visualized at the TEE imaging angle of 135°.

**Figure 1:**
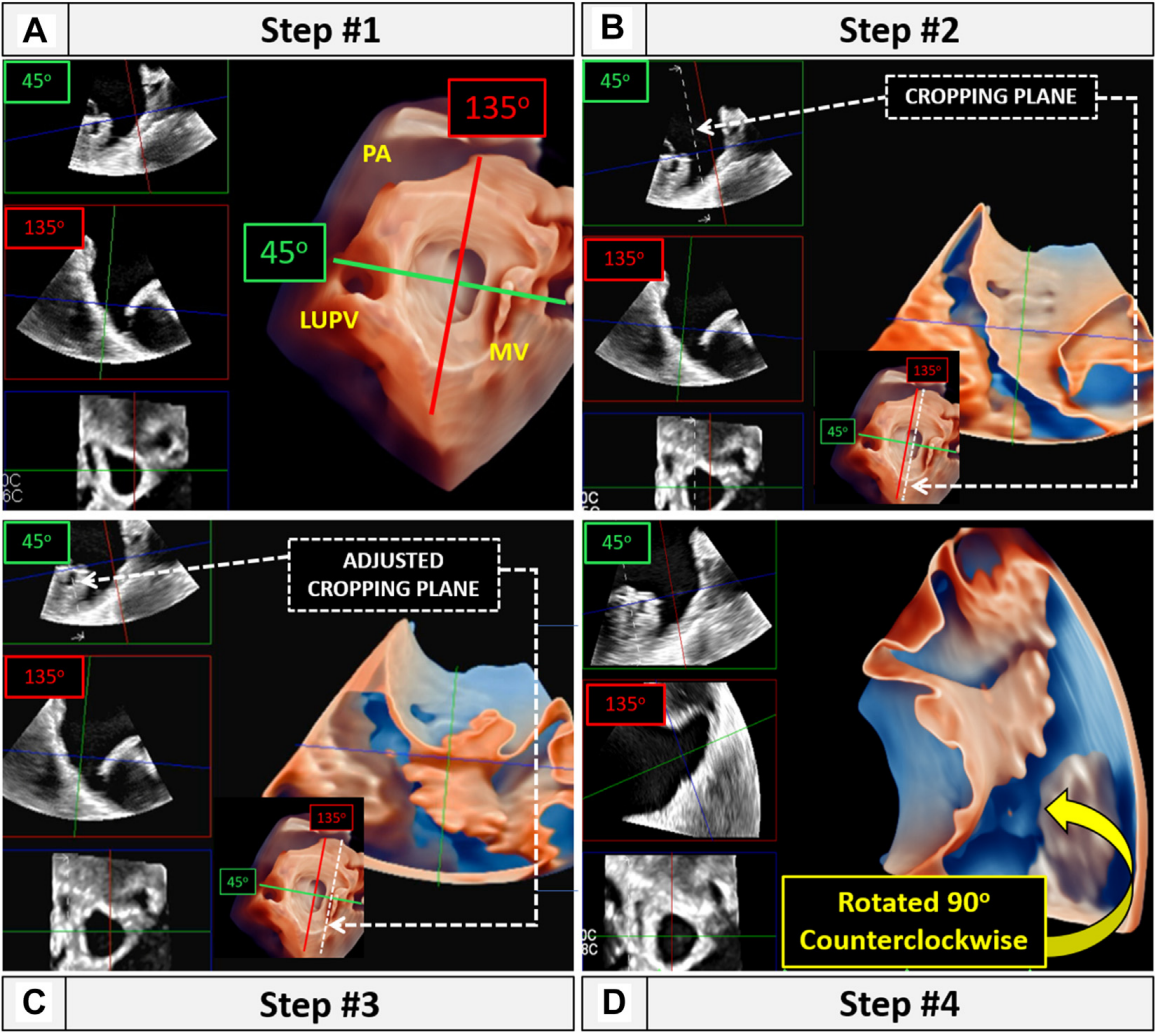
Steps of LAAM technique. (A) Three-dimensional midesophageal LAA en face view is obtained at 2D 45° angle (*green line*) with transillumination feature on. Orthogonal plane (*red line*) is then obtained using multiview. (B) Red reference plane is selected, and cropping plane (*dotted lines*) is demonstrated. (C) Cropping plane is adjusted leftward to uncrop the full 3D LAA anatomy. (D) Three-dimensional image is rotated 90° counterclockwise, simulating the standard fluoroscopic view during the percutaneous LAA occlusion procedures. *LUPV*, Left upper pulmonary vein; *MV*, mitral valve; *PA*, pulmonary artery.

## Step 1

At two-dimensional (2D) TEE midesophageal 45° imaging angle, obtain a 3D zoom view of LAA with transillumination and multiplane features on. We prefer the vertical 3-panel layout mode with planes representing 45°, 135°, and en face LAA orifice views.

## Step 2

Select the 135° 2D reference plane to reveal the cropped 3D view of the LAA.

## Step 3

Adjust the cutting plane leftward to reveal entire LAA.

## Step 4

Rotate image counterclockwise by 90° to simulate standard LAA occlusion fluoroscopic guidance view in the right anterior oblique and caudal projection.

## Disclosure statement

M.S. is on the Speakers Bureau of Abbott, Boston Scientific, Medtronic, and Philips and is on the Advisory Board of Siemens.

## Supplementary Data

Video 1 Steps of LAAM technique. (1) Three-dimensional midesophageal LAA en face view is obtained at 2D 45° angle (*green line*) with transillumination feature on. Orthogonal plane (*red line*) is then obtained using multiview. (2) Red reference plane is selected, and cropping plane (*dotted lines*) is demonstrated. (3) Cropping plane is adjusted leftward to uncrop the full 3D LAA anatomy. (4) Three-dimensional image is rotated 90° counterclockwise, simulating the standard fluoroscopic view during the percutaneous LAA occlusion procedures. *LUPV*, Left upper pulmonary vein; *MV*, mitral valve; *PA*, pulmonary artery.
